# The effect of polygenic risk score and childhood adversity on transdiagnostic symptom dimensions at first-episode psychosis: evidence for an affective pathway to psychosis

**DOI:** 10.1038/s41398-024-03149-7

**Published:** 2024-10-26

**Authors:** Luis Alameda, Victoria Rodriguez, Marta Di Forti, Edoardo Spinazzola, Giulia Trotta, Celso Arango, Manuel Arrojo, Miguel Bernardo, Julio Bobes, Lieuwe de Haan, Cristina Marta Del-Ben, Charlotte Gayer-Anderson, Lucia Sideli, Peter B. Jones, James B. Kirkbride, Caterina La Cascia, Giada Tripoli, Laura Ferraro, Daniele La Barbera, Antonio Lasalvia, Sarah Tosato, Pierre-Michel Llorca, Paulo Rossi Menezes, Jim van Os, Bart P. Rutten, Jose Luis Santos, Julio Sanjuán, Jean-Paul Selten, Andrei Szöke, Ilaria Tarricone, Andrea Tortelli, Eva Velthorst, Hannah E. Jongsma, Evangelos Vassos, Diego Quattrone, Robin M. Murray, Monica Aas

**Affiliations:** 1 Service of General Psychiatry, Treatment and Early Intervention in Psychosis Program, Lausanne University Hospital (CHUV), Lausanne, Switzerland; 2 Psychosis Studies, Institute of Psychiatry, Psychology and Neuroscience, King’s College of London, London, UK; 3 Instituto de Investigación Sanitaria de Sevilla, IbiS, Hospital Universitario Virgen del Rocío, Department of Psychiatry, Universidad de Sevilla, Sevilla, Spain; 4 Social, Genetics and Developmental Psychiatry Centre, Institute of Psychiatry, Psychology and Neuroscience, King’s College London, London, UK; 5 Department of Child and Adolescent Psychiatry, Institute of Psychiatry and Mental Health, Hospital General Universitario Gregorio Marañón, School of Medicine, Universidad Complutense, IiSGM, CIBERSAM, Madrid, Spain; 6 Department of Psychiatry, Psychiatric Genetic Group, Instituto de Investigación Sanitaria de Santiago de Compostela, Complejo Hospitalario Universitario de Santiago de Compostela, Santiago, Spain; 7 Barcelona Clinic Schizophrenia Unit, Hospital Clinic, Departament de Medicina, Institut de Neurociències (UBNeuro), Universitat de Barcelona (UB), Institut d’Investigacions Biomèdiques August Pi I Sunyer (IDIBAPS), CIBERSAM, ISCIII, Barcelona, Spain; 8 Instituto de Investigación Sanitaria del Principado de Asturias (ISPA), Oviedo, Spain; 9 Department of Medicine, Psychiatry Area, School of Medicine, Universidad de Oviedo, Centro de Investigación Biomédica en Red de Salud Mental (CIBERSAM), Oviedo, Spain; 10 Department of Psychiatry, Early Psychosis Section, Amsterdam UMC, University of Amsterdam, Amsterdam, The Netherlands; 11 Neuroscience and Behaviour Department, Ribeirão Preto Medical School, University of São Paulo, São Paulo, Brazil; 12 ESRC Centre for Society and Mental Health, King’s College London, London, UK; 13 Department of Human Science, LUMSA University, Rome, Italy; 14 Section of Psychiatry, Department of Biomedicine, Neuroscience and advanced Diagnostic (BiND), University of Palermo, Palermo, Italy; 15 Department of Psychiatry, University of Cambridge, Cambridge, UK; 16 CAMEO Early Intervention Service, Cambridgeshire & Peterborough NHS Foundation Trust, Cambridge, UK; 17 Psylife Group, Division of Psychiatry, University College London, London, UK; 18 Section of Psychiatry, Department of Neuroscience, Biomedicine and Movement, University of Verona, Verona, Italy; 19 Université Clermont Auvergne, Clermont-Ferrand, France; 20 Department of Preventive Medicine, Faculdade de Medicina, Universidade de São Paulo, São Paulo, Brazil; 21 Department of Psychiatry and Neuropsychology, School for Mental Health and Neuroscience, South Limburg Mental Health Research and Teaching Network, Maastricht University Medical Centre, Maastricht, The Netherlands; 22 Department Psychiatry, Brain Centre Rudolf Magnus, Utrecht University Medical Centre, Utrecht, The Netherlands; 23 Department of Psychiatry, Servicio de Psiquiatría Hospital “Virgen de la Luz”, C/Hermandad de Donantes de Sangre, 16002 Cuenca, Spain; 24 Department of Psychiatry, School of Medicine, Universidad de Valencia, Centro de Investigación Biomédica en Red de Salud Mental (CIBERSAM), C/Avda. Blasco Ibáñez 15, 46010 Valencia, Spain; 25 Rivierduinen Institute for Mental Health Care, Leiden, The Netherlands; 26 University of Paris Est Creteil, INSERM, IMRB, AP-HP, Hôpitaux Universitaires, H. Mondor, DMU IMPACT, Creteil, France; 27 Bologna Transcultural Psychosomatic Team (BoTPT), Department of Medical and Surgical Science, Alma Mater Studiorum Università di Bologna, Bologna, Italy; 28 Institut Mondor de recherché biomedicale, Creteil, France; 29 Department of Research, Community Mental Health Service, GGZ Noord-Holland-Noord, Heerhugowaard, The Netherlands; 30 Centre for Transcultural Psychiatry ‘Veldzicht’, Balkbrug, The Netherlands

**Keywords:** Schizophrenia, Clinical genetics

## Abstract

Childhood adversity is associated with various clinical dimensions in psychosis; however, how genetic vulnerability shapes the adversity-associated psychopathological signature is yet to be studied. We studied data of 583 First Episode Psychosis (FEP) cases from the EU-GEI FEP case-control study, including Polygenic risk scores for major depressive disorder (MDD-PRS), bipolar disorder (BD-PRS) and schizophrenia (SZ-PRS); childhood adversity measured with the total score of the Childhood Trauma Questionnaire (CTQ); and positive, negative, depressive and manic psychopathological domains from a factor model of transdiagnostic dimensions. Genes and environment interactions were explored as a departure from a multiplicative effect of PRSs and total CTQ on each dimension. Analyses were adjusted for age, sex, 10 PCA, site of recruitment and for medication. A childhood adversity and PRS multiplicative interaction was observed between A) the CTQ and MDD-PRS on the predominance of positive (β = 0.42, 95% CI = [0.155, 0.682], p = 0.004); and depressive (β = 0.33, 95% CI = [0.071, 0.591], p = 0.013) dimensions; B) between the CTQ and BD-PRS on the positive dimension (β = 0.45, 95% CI = [0.106, 0.798], p = 0.010), and C) with the CTQ and SZ-PRS on the positive dimension (β = −0.34, 95% CI = [−0.660, −0.015], p = 0.040). Bonferroni corrected p-value of significance was set at 0.0125. In conclusion, despite being underpowered, this study suggests that genetic liability for MDD and BD may have a moderating effect on the sensibility of childhood adversity on depressive and positive psychotic dimensions. This supports the hypothesis of an affective pathway to psychosis in those exposed to childhood adversity.

## Introduction

Childhood adversity is a well-known environmental risk factor for mental illness, conferring around a threefold increase in risk of both affective and psychotic disorders [1–3]. Childhood adversity is associated with worsening the psychopathological profile [4, 5] and the functioning of people with a psychotic disorder (PD) [6]. Despite some improvement in the understanding of the psychological processed involved, the biological mechanisms involving childhood adversity are poorly understood [7, 8].

The role of genes and their interplay with environmental risk (here called Gene & Environment Interplay, G&E) has developed strongly throughout the last 15 years, including recent studies exploring interaction of genetic liability in the form of polygenic risk score (PRS) with environmental risk factors. PRSs are calculated by using subsets of single-nucleotide polymorphisms (SNPs) from large case control genome-wide association studies. SNPs are selected according to their p-value and weighted by their effect size to calculate a PRS for each individual in an independent validation sample. The PRS can then be tested for its ability to differentiate between cases and controls in the validation dataset, and currently account for up to 24%, 20% and 9% of schizophrenia, bipolar disorder and depression variance respectively [9–11]. Current meta-analytical evidence so far shows a very small correlation between schizophrenia PRS (SZ-PRS) and childhood adversity exposure (gene and environment correlation) [12], and inconsistent results in terms of interaction [13]. To date, evidence is mostly based on general population samples, with only a handful of studies examining people with an actual PD [14–17]. Moreover, no study to date has examined the role of genetic liability in interaction with childhood adversity on specific psychopathological features of psychosis, which is an important gap. When exploring G&E interplay by using a broad category of First Episode of Psychosis (FEP) or PDs, more specific associations with psychotic symptom dimensions may be overlooked. This is a limitation as there is evidence pointing to the presence of specific effects between childhood adversity subtypes and clinical dimensions [5] that could be explained by distinct underlying mechanism. Furthermore, there is evidence that different clinical phenotypes within PDs, such as affective psychosis may be more strongly associated with PRS for depression (MDD-PRS) or for Bipolar disorder (BD-PRS), than to SZ-PRS; while non-affective psychosis is more strongly related to SZ-PRS [18]. Thus, using dimension specific PRSs associations within the psychosis spectrum could be a useful tool to explore more in depth the genetic underpinning of various adversity-related phenotypes in PDs.

Given the absence of studies exploring the G&E interplay in clinical dimensions of psychosis and given the suggestive evidence showing distinct association of various PRSs in differentiating clinical phenotypes in psychosis, we planned the current study. The objective is to test the G&E interaction between PRS for MDD, BD and SZ and childhood adversity for the predominance of positive, negative, depressive and manic dimensions calculated with a factor model. We hypothesize that (i) the genetic vulnerability of MDD will interact with childhood adversity influencing a clinical profile characterised by a more pronounced depressive dimension. (ii) genetic vulnerability of BD will interact with childhood adversity influencing a clinical profile characterised by a more pronounced manic dimension; (iii) that the genetic vulnerability of SCZ will interact with childhood adversity influencing a clinical profile characterised by a more pronounced positive and negative dimensions.

## Materials and methods

### Sample design and procedures

The current study uses a sample of FEP patients, part of the larger case-control study EUropean network of national schizophrenia networks studying Gene- Environment Interactions (EU-GEI) [19]. While we will describe the procedure of the main case-control study, only a subset of the cases will be part of the analyses here presented. FEP patients were identified between 2010 and 2015 across six countries to examine incidence rates of PD and patterns of symptomatology [19]. An extensive face-to-face assessment was conducted on 1130 FEP and 1497 controls, and DNA samples were collected for a subset of them (73.6% of the cases and 78.5% of the controls). Broadly, this sample was judged to be representative of the population living in each catchment area by age, sex and ethnic group as detailed in the Supplementary Material (SM) and previously shown [19, 20]. FEP patients were included in the case–control study if meeting the following criteria during the recruitment period: (a) age between 18 and 64 years; (b) presentation to the mental healthcare services with a clinical diagnosis of and minimally treated for a FEP; (c) residency within the catchment area. Exclusion criteria were: previous diagnosis and/or treatment for a psychotic disorder; a diagnosis of organic psychosis (ICD-10: F09); or transient psychotic symptoms resulting from acute intoxication (ICD- 10: F1X.5); language barriers.

Briefly, patients were identified by clinically trained researchers who carried out regular checks across the 17 catchment areas. The diagnosis was confirmed by the Operational Criteria Checklist for Psychotic and Affective Illness (OPCRIT) within the EU-GEI consortium [21, 22], which also allowed to create dimensional scores (details below and in SM). As described by Gayer-Anderson et al. [19], research teams included trained research nurses and clinical psychologists and were overseen by a psychiatrist with experience in epidemiological research. Full additional information from recruitment process and representativeness of the sample can be found in supplementary materials (SM).

### Ethics approval and consent to participate

Written informed consent was obtained from participants and institutional review board (IRB) approvals were granted from all centres. Ethical approval was provided from local research ethics committees in each catchment area: South London and Maudsley and Institute of Psychiatry Research Ethics Committee; National Research Ethics Service Committee East of England–East Cambridge; Medisch-Ethische Toetsingscommissie van het Academisch Centrum te Amsterdam; Comité Ético de Investigación Clínica Hospital Gregorio Marañón; Comité Ético de Investigación Clínica del Hospital Clinic de Barcelona; Comité Ético de Investigación Clínica del Hospital Clinic Universitari de Valencia; Comité Ética de la Investigación Clínica del Principado de Asturias; Comité Ético de Investigación Clínica de Galicia; Comité Ético de Investigación Clínica del Hospital Virgen de la Luz de Cuenca; Comité de Protéction des Personnes–CPP Île de France IX; Comitato Etico Policlinico S Orsola Malpighi; Comitato Etico Azienda Ospedaleria Universitaria di Verona; Comitato Etico Palermo 1, Azienda Ospedaliera Policlinico “Paolo Giaccone”; and Research Ethics Committee of the clinical Hospital of Ribeirão Preto Medical School, University of São Paulo, Brazil. All methods were performed in accordance with the relevant guidelines and regulations. Authors have obtained written informed consent for publication of the images involving data from the EUGEI study [23].

### Measures

#### Socio-demographic characteristics

Socio-demographic data were collected using the Medical Research Council (MRC) Socio-demographic Schedule modified version [24], and supplemented with additional information from medical records on educational attainment, employment, marital and living status. Ethnicity was self-ascribed using categories employed by the 2001 UK Census (http://www.ons.gov.uk/ons/guide-method/census/census-2001/index.html), but genetic ancestry (ethnicity genetically driven) was also created using Principal Component Analyses (PCA, see below).

#### Childhood Trauma Questionnaire (CTQ)

CTQ [25, 26] was used to measure the exposure to past experiences of abuse (sexual, physical, and emotional) and neglect (physical and emotional). This self-report instrument contains 28 questions covering multiple questions enquiring on experiences of abuse and neglect occurring prior to the age of 18, with answers ranging from ‘never true’, through ‘rarely true’, ‘sometimes true’, ‘often true’, to ‘very true’. Given our limited sample size not allowing the exploration of specific subtypes, a broad composite measure of CTQ total score was calculated by adding up the trauma scores to a total score (ranging from 0 - no exposure-, up to 140 - maximum possible score-). A recent review shows that is the most commonly used instrument in this field [27].

#### OPCRIT and Diagnostic procedure

We made DSM-IV diagnoses [28] from interviews and mental health records utilizing the Operational Criteria Checklist (OPCRIT) at baseline [22] by centrally trained investigators, whose reliability was assessed throughout the study (κ = 0.7), as detailed in SM. In brief, OPCRIT is semi-structured checklist including 92 items covering mental health clinical background and psychopathology. These derived diagnoses were grouped into Schizophrenia Spectrum Disorder (SSD) (or non-affective disorders) group (codes 295.1–295.9 and 297.1–298.9) or Affective Disorder (AP) group (patients diagnosed with codes 296–296.9), and which included patients with bipolar disorder (BD) (codes 296.0–296.06 and 296.4–296.89) and major depression with psychotic features (MDD-P, codes 296.2–296.36).

#### Bi-factor dimensional models of psychopathology

Beside the creation of the diagnostic categories, OPCRIT data was also used to delineate the psychopathological dimensions. Using item response modelling in M*plus*, version 7.4, two separate bi-factor models of psychopathology were estimated based on the associations among ratings of psychotic symptoms in patients and controls which were dichotomised as 0 ‘*absent’* or 1 ‘*present’*. This methodology is described in full in earlier EU-GEI papers on transdiagnostic dimensions [21] and it has been used in other EU-GEI publications [29, 30]. Briefly, the estimation of the five specific symptom dimensions’ scores (positive, negative, disorganisation, mania and depression) were available alongside a general dimension, and were derived from a bifactor model built using multidimensional item response modelling from the 59 items describing psychopathology in OPCRIT. Various model fit statistics were used to prove the reliability and strength of the model proving the validity of the bi-factor model used [21] and statistical details about the model is described in full in a previous study [21]. These scores reflect how psychopathological items aggregate contributing to particular factors and should be interpreted as a predominance of a particular dimensional profile, and not symptoms severity such as that of the Positive and Negative Syndrome Scale (PANSS) [31].

In the current study we focused on the positive, negative, depressive and manic dimensions in line with the available PRS of SZ, MDD and BD respectively.

#### Genotyping

DNA from blood or saliva were obtained at baseline in 73.6% of cases from the entire EUGEI-Work package two sample [19]. The DNA collected was genotyped at the MRC Centre for Neuropsychiatric Genetics and Genomics in Cardiff (UK) using a custom Illumina HumanCoreExome-24 BeadChip genotyping array, with quality control performed locally as previously described [32]. A PCA generating 10 principal components (PC) was run on pruned variants to control for population stratification as detailed in the SM. Following the same procedures previously published [32], PRS for SZ, BD and depression (SZ-PRS, BD-PRS and MDD-PRS) were built on PRSice2 [33] using data from the largest GWAS available [11, 34, 35], at the p-value threshold 0.05 that better predicted most phenotypes in the original GWAS publications. Each PRS was standardized to a mean of zero and standard deviation of one.

As previously described in previous papers by our group [18], one of the limitations when building current PRSs, which are derived from mostly European samples, is their reduced predictive power in multi-ethnic samples [36]. This has been shown in a previous study on FEP patients [37], where PRS-SZ had much lower predictive power in the African ancestry population. In light with this, we constrained the sample to those categorised as of European ancestry based on PCA (details provided in SM) for the scope of the present study.

### Statistical analyses

Only participants who had complete data on PRS, childhood adversity using the CTQ, OPCRIT data and identified as white European ancestry based on PCA data were included for the analyses of this article. Comparisons between FEP participants that were included and those excluded were examined (Table S1, SM).

We described sociodemographic using frequencies, percentages, mean and standard deviations (SD) using Chi-square and Student t test as appropriate. Interactions between CTQ and PRSs (MDD-PRS, BD-PRS and SZ-PRS) on symptom dimensions were explored as a departure from a multiplicative effect. More specifically, we run four independent linear regression models with each dimension as dependent variable, including the three PRSs, the CTQ total score, and its product with each PRS; adjusted for sex, age, site, 10 PCs and the interaction with each PRS and total CTQ score [38, 39]. Assumption for regression analyses were also checked and found satisfying. To control for multiple testing (one model per dimension therefore four tests), Bonferroni adjusted p-value was set at <0.0125 (0.05/4 = 0.0125). p-values between 0.0125 and 0.05 were considered as trend level in the reported results. We conducted a series of sensitivity analyses to account for medication effect consisting of comparison of mean CTQ scores in those taking four main types of drugs (benzodiazepines, antipsychotics, antidepressants and mood stabilisers) and those being medication free in order to understand whether people with high adveristy scores tended to be more represented among those taking medication. Whenever CTQ scores were different according to exposure to any medication, further sensitivity analyses were conducted adjusting each main model by medication use in addition to the primary analyses. Analyses were conducted using Statistical Package for Social Sciences, Version 26.0 (SPSS Inc) and STATA Software.

## Results

### Demographic overview of the sample

The sample characteristics of the final analyzed sample of 583 FEP patients are shown in Table 1 (flow chart with detail on exclusions are in SM), which proved to be similar to those FEP not included in terms of age and sex but tended to have fewer years of education (*p* < 0.001), lower CTQ total scores (*p* = 0.01) and were more often diagnosed with psychotic depression (*p* = 0.02) (SM Table S1). The mean age of the included patients was 31.79 ± 10.96, 63% of them were male. The mean years of education was 12.86 ± 4.02; with diagnoses distributed as 43% of schizophrenia, 4.3% schizoaffective disorder, 12% bipolar disorder with psychotic features, 14.6% major depression with psychotic features and 25% had other diagnoses (i.e. brief psychotic episode, schizophreniform disorder or psychosis non otherwise specified).

**Table 1:** Demographics (*N* = 583).

Age, mean ± SD	31.79 ± 10.96
Years of education mean ± SD	12.86 ± 4.02
CTQ total, mean ± SD	41.34 ± 13.28
DSM Diagnosis	
Schizophrenia, N (%)	253 (43.4)
Other Psychosis, N (%)	150 (25.7)
Schizoaffective Disorder, N (%)	25 (4.3)
Bipolar disorder, N (%)	70 (12.0)
Psychotic depression, N (%)	85 (14.6)
Sex, Males N (%)	369 (63.3)
Antidepressants Yes (%)	129 (22.1)
Antipsychotics Yes (%)	444 (76.2)
Mood stabilizers Yes (%)	77 (13.2)
Benzodiazepine Yes (%)	220 (37.7)

Medication: 517 of the 583 patients included in the study had data on antipsychotic medication, mood stabilizers and benzodiazepines. All patients had data on antidepressants. All participants were classified as European based on their Principal Component Analyses.

### Childhood adversity scores and symptom dimensions

No significant associations were found between CTQ total score and the symptom dimensions (positive; β = −0.05, 95% CI = [−0.168, 0.072], *p* = 0.466; negative β = 0.00, 95% CI = [−0.117, 0.119], *p* = 0.990; manic β = 0.05, 95% CI = [−0.071, 0.174] *p* = 0.441 or depressive β = 0.09; 95% CI = [−0.018, 0.218], *p* = 0.099).

### Depression polygenic risk score, childhood adversity and symptom dimensions

**Fig. 1:**
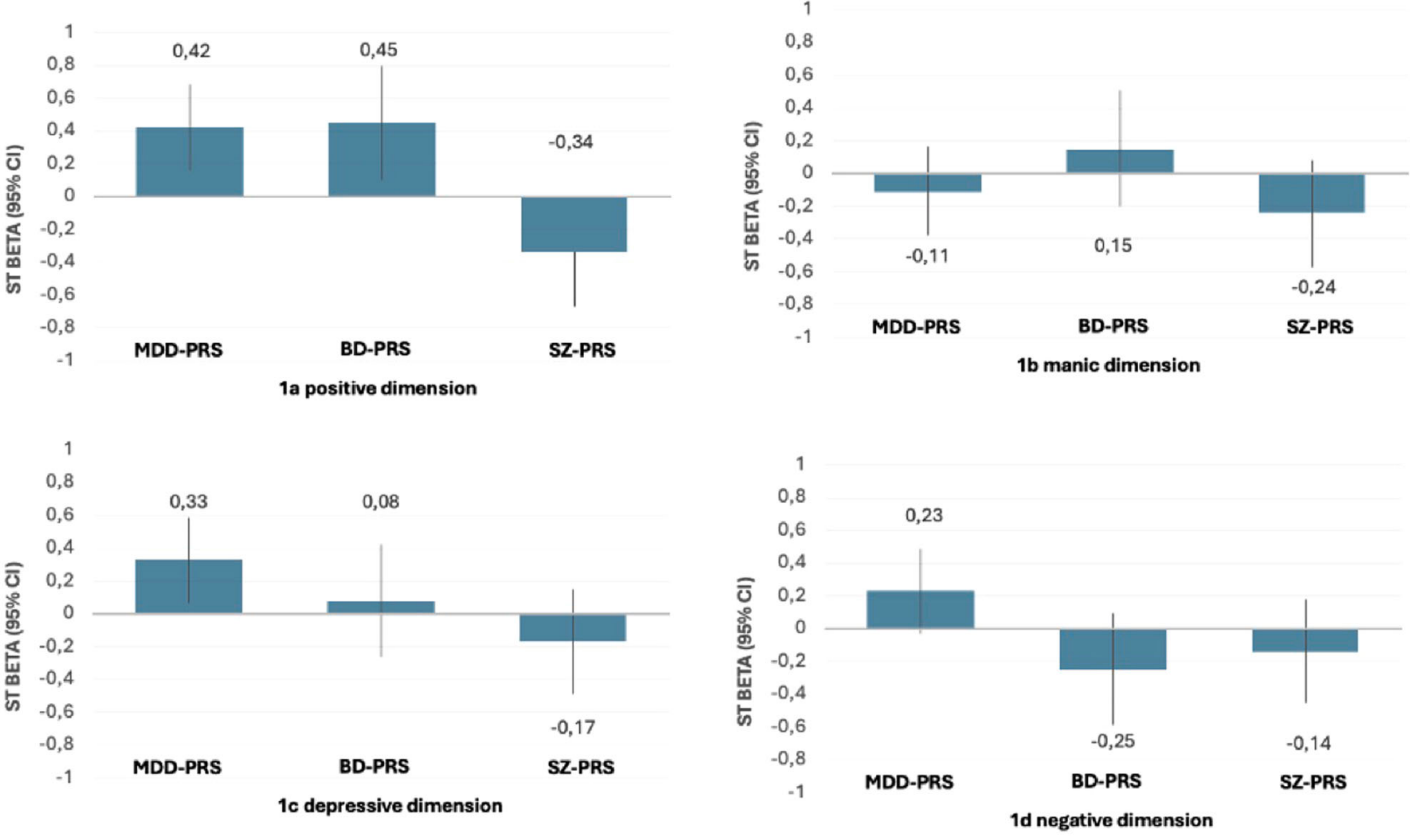
Multiplicative interaction effects of childhood adversity and polygenic risk scores on psychopathological dimensions. **a** Positive dimension, childhood adversity & PRS (MDD, BD, SZ) interactions. **b** Manic dimension childhood adversity & PRS (MDD, BD, SZ) interactions. **c** Depressive dimension, childhood adversity & (MDD, BD, SZ) interactions. **d** Negative dimension, childhood adversity & PRS (MDD, BD, SZ) interactions. This figure illustrates the magnitude of the multiplicative interaction effect of PRS of depression (MDD-PRS), bipolar disorder (BD-PRS) and schizophrenia (SZ-PRS) with adversity (measured with the Childhood Trauma Questionnaire total score). An interaction effect into the positive direction can be interpreted as an increased sensibility of adversity exposure on a dimension, moderated by genetic vulnerability.

MDD-PRS showed a significant association with the positive dimension (β = −0.48; 95% CI = [−0.765, −0.200], *p* = 0.002), and the negative dimension (β = −0.48; 95% CI = [−0.754, −0.199], *p* = 0.002); but was not associated with the manic dimension (β = 0.13, 95% CI = [−0.163, 0.417], *p* = 0.422) or the depressive dimension (β = −0.12, 95% CI = [−0.397, 0.163], *p* = 0.445). At the level of interactions, a significant positive interaction was observed between childhood adversity and MDD-PRS on the positive dimension (β = 0.42, 95% CI = [0.155, 0.682], *p* = 0.004) 9 (Fig. 1a). Although non-significant (according to Bonferroni adjusted p-value of 0.0125), a trend level positive interaction was observed between childhood adversity and MDD-PRS on the predominance of depressive dimension (β = 0.33, 95% CI = [−0.071, 0.591], *p* = 0.020) (Fig. 1b). No interaction effects were found for the manic and the negative dimensions between MDD-PRS and CTQ (Table 2 and Fig. 1).

**Table 2:** OPCRIT dimensions, polygenic risk score (PRS) and trauma.

CTQ, Total score	Positive dimension		Manic dimension		Depressive dimension		Negative dimension	
	ß (95% CI)_	*P*	ß (95% CI)	*p*	ß (95% CI)	*p*	ß (95% CI)	*p*
**MDD-PRS** Interaction	**0.42 (0.155, 0.682)**	**0.004**	−0.11, (−0.383, 0.158)	0.446	**0.33, (0.071, 0.591)**	**0.013**	0.23, (−0.025, 0.495)	0.099
**BD-PRS** Interaction	**0.45, (0.106, 0.798)**	**0.010**	0.15, (−0.209, 0.500)	0.452	0.08, (−0.262, 0.423)	0.667	−0.25, (−0.593, 0.089)	0.177
**SZ-PRS** Interaction	−**0.34 (**−**0.660**, −**0.015)**	**0.040**	−0.24, (−0.564, 0.094)	0.191	−0.17, (−0.491, 0.145)	0.320	−0.14 (−0.459, 0.175)	0.412

Adjusted for age, sex, site, 10 PCA, and country. *MDD-PRS* Major depressive Disorder Polygenic Risk Score, *BD-PRS* Bipolar Disorder Polygenic Risk Score, *SCZ-PRS* Schizophrenia Polygenic Risk Score. Bold = trend level significance levels in relation to the Bonferroni corrected p-value set at 0.0125.

### Bipolar disorder polygenic risk score, childhood adversity and symptom dimensions

BD-PRS showed a trend-level association with the positive dimension (β = −0.49, 95% CI = [−0.875, −0.102], *p* = 0.021) but no association with the negative (β = 0.09, 95% CI = [−0.288, 0.474], *p* = 0.654), manic (β = −0.19, 95% CI = [−0.582, 0.208], *p* = 0.387) or depressive dimensions (β = −0.20, 95% CI = [−0.585, 0.178], *p* = 0.330). At the level of interactions, a significant positive interaction was found between childhood adversity and BD-PRS on the predominance of the positive dimension (β = 0.45, 95% CI = [0.106, 0.798], *p* = 0.010). No interaction effects were found for the negative manic or depressive dimensions with BD-PRS and CTQ (Table 2 and Fig. 1).

### Schizophrenia polygenic risk score, childhood adversity and symptom dimensions

SCZ-PRS showed a non-significant trend association with the positive dimension (β = 0.35, 95% CI = [−0.005, 0.723], *p* = 0.053) and with the manic dimension (β = 0.37, 95% CI = [0.003, 0.745], *p* = 0.048), while no association was found with the negative (β = 0.19, 95% CI = [−0.165, 0.551], *p* = 0.291), or the depressive dimension (β = −0.10, 95% CI = [−460, 0.259], *p* = 0.608). At the level of interactions, a trend-level negative interaction was found between childhood adversity and SZ-PRS on the predominance of the positive dimension (β = −0.34, 95% CI = [−0.660, −0.015], *p* = 0.040). No interaction effects were found for the manic, the depressive or negative dimensions with SZ-PRS and CTQ (Table 2 and Fig. 1).

### Sensitivity analyses adjusted by medication

Mean CTQ scores by medication status (being currently medicated or not) for various medication (antipsychotics, benzodiazepines, mood stabilized and antidepressants) are shown in SM (Table S2). In brief, these analyses showed that patients taking antipsychotics tended to have higher scores of CTQ, although this was not significant when adjusted by multiple correction (*p* = 0.020; multiple corrected p-value set at 0.012). Nevertheless, we conducted the interaction analyses also adjusting for antipsychotic medication with effect sizes and p-values changing only marginally (details can be found in Table S3 in SM), allowing to conclude that medication was not a source of confounding. .

## Discussion

This is the first study in FEP examining the interaction between childhood adversity and PRSs of SZ, BD and MDD in relation to clinical dimensions. To the best of our knowledge this has never been done in other psychiatric disorders either. Our main findings suggest a relationship between childhood adversity and positive and depressive symptoms of psychosis moderated by the polygenic load for major mental illnesses. In line with our hypothesis, we found that those with a high MDD-PRS and with a history of childhood adversity were more likely to have a clinical profile characterised by the depressive symptoms (ß = 0.33). Interestingly, this was also the case for positive dimensions (ß = 0.42). On the other hand, no evidence was found to support our hypotheses that a genetic vulnerability for BD and exposure to childhood adversity would moderate the sensibility of childhood adversity on the expression of manic symptoms; rather BP-PRS seemed also moderate the expression of positive symptoms of psychosis in those with childhood adversity (ß = 0.45). These two findings support an affective pathway to psychosis where genetic vulnerability for depression and bipolar disorder influence the positive symptoms domain of psychosis. Unexpectedly, SZ-PRS and childhood adversity interactions did not play role in the predominance of positive dimension (ß = −0.34).

As stated above, our findings shed new light on the “Affective pathway to psychosis” hypothesis, showing that MDD and BD PRS interact with CTQ on the predominance of a clinical profile characterised by positive symptoms. This model usually focuses on mediation rather than moderation and postulates that low mood and other mood related attributes such as anxiety, are mediators between social adversity and psychosis [7, 40–42]. To the best of our knowledge this is the first study to explore whether genetic vulnerability for depression moderates the effect of childhood adversity on psychopathology in patients with FEP. This supports that depressive mood may not only be a mediator, but also that at a genetic level, vulnerability for depression moderates the adversity-psychosis dyad. There is evidence suggesting that low level depressive symptoms (at a sub-diagnostic level) are important for the aetiopathogenesis of psychosis and outcome, as they contributes to impairment in reality testing therefore contributing to the emergence of low level symptoms of psychosis [43] and psychosis onset [43]. Furthermore, subclinical depressive symptoms often precede relapse during the course of psychosis and play a role in functional outcomes [44, 45] and of course suicidality [43, 46]. Beyond the suggested importance of assessing subthreshold depressive symptoms for these reasons, our findings also suggest that considering genetic load for depression may be a marker for a more severe psychopathological profile in people with psychosis and trauma. Of course, the currently availably PRSs still explain a relatively small proportion of the variance and therefore more powerful MDD and BD GWAS are needed to allow translating their biological importance into a useful biomarker for clinical practice [47].

Our findings on the negative interaction found for childhood adversity with SZ- PRS on positive symptoms should be interpreted cautiously as results did not survive to Bonferroni correction. A possible explanation is that the influence of childhood adversity on the positive domain for those with a high genetic vulnerability schizophrenia is low due to a “ceiling effects”; while the role of adversity have more relative weigh in those of a lower genetic risk. In a recently published meta-analysis on various adversities and psychopathological dimensions in psychosis we found that neglect, but not abuse, is associated with more severe positive symptoms [5]; with our current findings suggesting that that association seems to not be moderated by genetic vulnerability.

### Study limitations and methodological considerations

Childhood adversity was reported retrospectively, with the inherent weakness of the retrospective design when examining traumatic events in people with psychosis due to recall biais [48]. However, a recent study suggests that that subjective reports of exposure to trauma are relevant for cognition impairment, therefore supporting the use of retrospective self-reports [49]. Secondly, the CTQ does not cover all types of childhood adversities such as bullying. Third, the number of cases included was relatively small for the number of comparisons we conducted, which may have limited the statistical power and made some of our finding nonsignificant. Although we interpreted the findings based on the direction and magnitude of the associations and interactions, overall, it is essential to point out that the study is underpowered and that larger studies on the topic are needed to confirm our claims, that remain tentative. Lastly, it should be noted that the OPCRIT scores built with a factorial analysis are not intended to represent a severity score, such as that of the PANSS or similar scales [31], but as a score that reflects how psychopathological items aggregate contributing to particular factors. The scores we use should be interpreted as a “predominance” of a particular dimensional profile, and not the severity of those symptoms, therefore comparisons with studies that use a severity score is not possible. For example, none of our analyses on CTQ and the dimensions were significant, which means that CTQ (in the absence of genetic vulnerability) is not associated with the predominance of any specific clinical domain in our sample. This is not line with previous work examining the association between trauma and severity of symptoms [5]. This lack of association could be explained by the fact that as our dimensional scores capture the “predominance” of one dimension over the others, and the role of trauma measured with a composite category is broad and transdiagnostic (influences in similar ways various symptoms). Those considerations aside, we believe this approach has great value because symptoms do not occur in isolation and tend to aggregate (for example delusions often co-occur with hallucinations), therefore it can be seen as a more realistic representation of the various clinical pictures of the heterogeneous psychosis phenotype.

In conclusion, our findings suggest that genetic liability for MDD and for BD have a interactive effect on the relationship between childhood adversity and positive psychotic symptom dimension, supporting the idea of an affective pathway to psychosis in those exposed to childhood adversity.

## Supplementary information

Suplementary Material

## Data Availability

The data set used for this study as well as the code for the analyses conducted is available upon request, subject to a data-sharing agreement with the EUGEI WP2 group.
