# Supplementary material for: The effect of polygenic risk score and childhood adversity on transdiagnostic symptom dimensions at first-episode psychosis: evidence for an affective pathway to psychosis
Source: Transl Psychiatry. 2024 Oct 26;14:454. doi: 10.1038/s41398-024-03149-7 (PMC11513137; doi:10.1038/s41398-024-03149-7)
Supplement: Supplementary file 1 — Suplementary Material [file 41398_2024_3149_MOESM1_ESM.docx]

**Supplementary Material**

**INDEX**

1. Table S1. Comparisons between subjects included in this study and those excluded (EUGEI patients Work package 2)

2. Flow chart

3. Examination of OPCRIT inter-rater reliability and measurement invariance

4. Additional information from recruitment process and representativeness of the sample

5. PCA analyses and definition of European ancestry based on principal components

6. Sensitivity analyses on CTQ and medication. Table S2

**Table S1. Comparisons between subjects included in this study and those excluded (EUGEI patients Work package 2)**

Table S1. Included and not included subjects (EUGEI patients)

|  | Included in the study  N=583 | Not includes  N=551 | Statistics |
| --- | --- | --- | --- |
| Age, mean±SD | 31.79±10.96 | 30.66±10.17 | T=178, DF=1132, p=.08 |
| Years of education mean±SD | 12.86±4.02 | 15.36±14.46 | T=-3.88, DF=1114,p<.001 |
| CTQ total, mean±SD | 41.34±13.28 | 43.73±15.52 | T=-2.55, DF=1003, p=.01 |
| DSM Diagnosis  Schizophrenia, N (%)  Other Psychosis, N (%)  Schizoaffective Disorder, N (%)  Bipolar disorder, N (%)  Psychotic depression, N (%) | 253 (43.4)  150 (25.7)  25 (4.3)  70 (12.0)  85 (14.6) | 234 (42.5)  183 (33.2)  22 (4.0)  60 (10.9)  52 (9.4) | X^2^=12.03, DF=4, p=.02 |
| Sex, Males N (%) | 369 (63.3) | 331 (60.1) | X^2^=1.24, DF=1, p=.27 |
| Antidepressants Yes (%)  Antipsychotics Yes (%)  Mood stabilizers Yes (%)  Benzodiazepine Yes (%) | 129 (22.1)  444 (76.2)  77 (13.2)  220 (37.7) | 85 (15.4)  452 (82.0)  80 (14.5)  200 (36.3) | X^2^=7.98, DF=1, p=.01  X^2^=1.74, DF=1, p=.19  X^2^=.13, DF=1, p=.73  X^2^=1.18, DF=1, p=.28 |

517 of the 583 patients included in the study had data on antipsychotic medication, mood stabilizers and benzodiazepine. 510 of the 551 patients not included in the study had data on antipsychotics, mood stabilizers and benzodiazepine. All patients included had data on antidepressants and 547 of 551 of the non-included patients had data available on antidepressants.

**2. Flow chart**


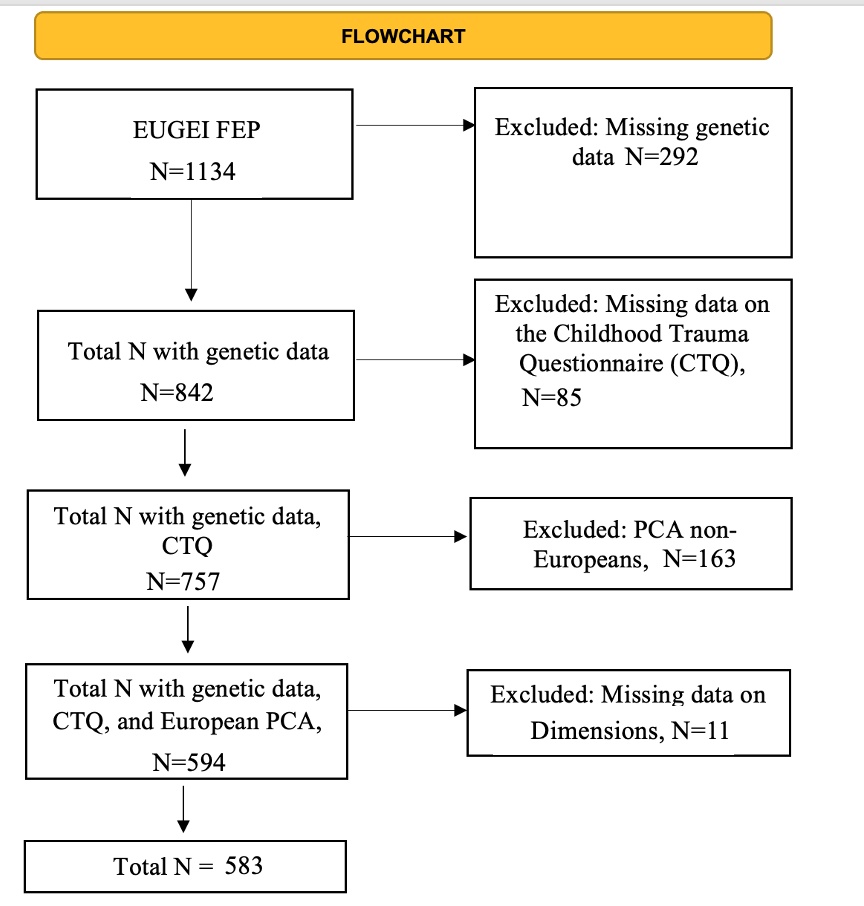


**3. Examination of cross-national OPCRIT inter-rater reliability and measurement invariance**

The OPerational CRITeria (OPCRIT) system^1, 2^ was used to: (1) assess

pre-morbid history and current mental state at FEP; and (2) establish a research based standardised diagnosis of psychotic disorder. The OPCRIT consists of a checklist that can be filled using different sources, e.g. case records or clinical interviews. Investigators’ training and monitoring was organised centrally on an online platform, which served to: implement and follow standardised procedures; provide psychopathology training; conduct all site inter-rater reliability pre-^3^ and post-training; and monitor the interrater reliability annually during the study^4^. All raters were included in central interrater reliability computations (k= 0.7). Cross-national OPCRIT inter-rater reliability investigators’ training and monitoring was organized centrally on an online platform, which served to: implement and follow standardized procedures; provide psychopathology training; conduct all-site inter-rater reliability pre- and post- training^3^; and monitor the inter-rater reliability annually during the study^5^. All raters were included in central interrater reliability computations. In addition, post-training inter-rater reliability for individual OPCRIT items within countries were calculated, finding the following values: United Kingdom, K-alpha 0.9085, 95% CI 0.8191 – 0.9760. The Netherlands, K-alpha 0.7770, 95% CI 0.7311 – 0.8205. France, K-alpha 0.7711, 95% CI 0.5739 – 0.9297. Spain, K-alpha 0.7679, 95% CI 0.6550 – 0.8495. Italy, K-alpha 0.5274, 95% CI 0.4926 – 0.5607. Noteworthy, the above agreement statistics refer to the OPCRIT item level, which are usually lower than the OPCRIT diagnostic level^6, 7^. Inter-rater reliability for Brazil could not be calculated, as only one rater in Brazil completed the central training and was in charge of OPCRIT administration. The lower interrater K-alpha for Italy may be due to the higher number of Italian raters involved in OPCRIT training and administration compared with other countries. However, this moderate value is in line with other studies. A review on OPCRIT reliability showed that, in six studies that provided the agreement between raters at the OPCRIT individual item level, the mean inter-rater kappa was 0.69 with a range from 0.48 to 0.84^6, 7^. Nevertheless, we adjusted our findings by country to take into account this disparity.

**4. Additional information from recruitment process and representativeness of the sample**

Here we provide details on the recruitment process and representativeness of the sample of the large EUGEI case control study from which the current study is a subsample in FEP only. As previously described in Di Forti et al., 2019^8^, recruitment process followed procedures previously used to generate representative samples of first episode psychosis (FEP) patients^9^ . The study includes individuals aged 18 to 64 years, who contacted mental health services for a suspected FEP, over periods up to four years in 17 catchment areas in England (Southeast London, Cambridgeshire & Peterborough); France (20th arrondissement of Paris, Val-de-Marne, Puy-de-Dôme); the Netherlands (Central Amsterdam, Gouda & Voorhout); Italy (part of the Veneto region, Bologna, and Palermo); Spain (Madrid-Vallecas, Barcelona, Valencia, Oviedo, Santiago, Cuenca), and; Brazil (Ribeirão Preto, Sao Paulo) (full details of the incidence sample recruitment and general description of the incidence study methods are available from the recently published paper by Jogsma et al 2018^10^). Case ascertainment involved trained researchers making regular contact with all secondary and tertiary mental healthcare providers to identify potential cases and searching electronic clinical records, where available. In this process, all cases with psychosis within services were considered. In all countries, it was uncommon for people to be treated for FEP in primary care; instead people with suspected psychosis would typically be referred to specialist mental health services. Research teams were overseen by a psychiatrist with experience in epidemiological research, and included trained research nurses and clinical psychologists. Teams received training in epidemiological principles and incidence study design to minimize non-differential ascertainment bias across different local and national healthcare systems. Between May 1, 2010, and April 1 2015, 1519 patients with FEP were approached. Of these 356 (1%) refused to participate, 19 (23%) could not consent because of language barriers and 14 (0·9%) were later excluded (London N=3; Madrid N=2; Bologna N=1; Ribeirão Preto N=8) as they did not meet the age inclusion criteria. For all patients who were not part of the study, local research ethics committees approved the extraction of demographics and clinical information from patient records. Patients who refused to participate were older [FEPconsented mean age=30·8 (10.5), median=29.0 (22.0 to 37.0); FEP refused mean age=32·8 (11·5), median=31.0 (25.0 to 42.0); p=0·0015], more likely to be women [FEPconsented male=558 (61·9%); FEP refused male=311 (54·7%), χ2(1)=7·6; p=0·0063] and of White European origin [χ2(5)=38, p<0·0001. 1130 FEP across the study sites consented to take part in the case-control study. The FEP recruited in the case-control study are broadly representative for gender and ethnicity of the rest of the incidence sample. However, in London, Amsterdam and Ribeirao Preto cases aged 18–24 were over-represented in the case-control sample and those aged 45–54 and 55 or over were under-represented compared with the incidence sample (full details in Jogsma et al ., 2018^10^.

**5. Principal components analysis and definition of European ancestry based on principal components**

As previously described^11^, principal components were calculated in PLINK using LD pruned variants across the whole sample. We ranked the sample in centiles based on Principal component 1 (PC1), and calculated proportion of self-reported European ethnicity in a dichotomy fashion (yes/no) on each centile. We established the cut-off point in the stacked PC1 when three groups in a row reported less than 0.5 of whiteness. We repeated the process for PC2 and used the two cut-off point as threshold in which those who fell within them were considered as European.

**6. Sensitivity analyses on CTQ and medication**

We calculated in FEP cases with available data on medication, the scores of CTQ between those taking medication (antidepressants, antipsychotics, mood stabilisers and benzodiazepines) and not taking medication. Given that we conducted four comparisons, the Bonferroni adjusted p-value was set at 0.125 (0.05/4 comparisons=0.0125) as can be seen below, none of the comparisons yielded any significant difference in CTQ score between FEP taking medication or not.

**Table S2**

|  | | | | | |
| --- | --- | --- | --- | --- | --- |
|  | Antidepressants | N | Mean | Std. Deviation | Std. Error Mean |
| ctq_tot | 1 | 129 | 42.15 | 14.537 | 1.28 |
|  | 0 | 454 | 41.12 | 12.901 | .605 |

t : 0.778; df: 581; Two-Sided p : 0.4370

|  | | | | | |
| --- | --- | --- | --- | --- | --- |
|  | Antipsychotics | N | Mean | Std. Deviation | Std. Error Mean |
| ctq_tot | 1 | 444 | 40.78 | 12.857 | .61039 |
|  | 0 | 73 | 44.67 | 15.425 | 1.805 |

t: -2.3251; df: 515; Two-Sided p : 0.0205

|  | | | | | |
| --- | --- | --- | --- | --- | --- |
|  | Mood stabilisers | N | Mean | Std. Deviation | Std. Error Mean |
| ctq_tot | 1 | 77 | 41.91 | 13.357 | 1.522 |
|  | 0 | 40 | 41.23 | 13.306 | .634 |

t: 0.4132; df: 515; Two-Sided p: 0.679

|  | | | | | |
| --- | --- | --- | --- | --- | --- |
|  | Benzodiazepines | N | Mean | Std. Deviation | Std. Error Mean |
| ctq_tot | 1 | 220 | 42.08 | 13.5 | .910 |
|  | 0 | 297 | 40.78 | 13.15 | .763 |

t: 1.0984; df: 515; Two-Sided p: 0.2725

Because the CTQ scores seemed to be higher in FEP taking antipsychotics showed some trend level non-significant trend Bonferroni corrected (p=0.0205) we conducted the interaction analyses adjusting for medication in the subsample of FEP with available data on medication (517 out of the 583 from the primary analyses). Results ca be found in Table S3 below.

**Table S3**

| **CTQ, Total score** | | **Positive dimension** | | **Manic dimension** | | **Depressive dimension** | | **Negative dimension** | |
| --- | --- | --- | --- | --- | --- | --- | --- | --- | --- |
|  | |  |  |  |  |  |  |  |  |
|  | |  |  |  |  |  |  |  |  |
|  | | ß (95% CI)_ | ***P*** | ß (95% CI) | ***p*** | ß (95% CI) | ***p*** | ß (95% CI) | ***p*** |
|  |  |  |  |  |  |  |  |  |  |
| **MDD-PRS**  Interaction |  | **.53 (.249, .801)** | **.001** | -.10, (-.387, .196) | .551 | **.27, (.001, .551)** | **.051** | .21, (-.064, .484) | .165 |
|  |  | | | | | | | | |
| **BD-PRS**  Interaction |  | **.38, (.176, .758)** | **.040** | .20, (-.184, .591) | .341 | -.03, (-.397, .339) | .886 | -.27, (-.630, .098) | .186 |
|  |  |  |  |  |  |  |  |  |  |
| **SZ-PRS**  Interaction |  | **-.30 (-.641, .040)** | **.084** | -.30, (-.659, .051) | .122 | -.14, (-.475, .201) | .462 | -0.20 (-.533, .137) | .285 |
|  |  | | | | | | | | |

**References**

1. Williams J, Farmer A, Ackenheil M, Kaufmann C, McGuffin P, Group ORR. A multicentre inter-rater reliability study using the OPCRIT computerized diagnostic system. *Psychological medicine* 1996; **26**(4)**:** 775-783.

2. McGuffin P, Farmer A, Harvey I. A polydiagnostic application of operational criteria in studies of psychotic illness: development and reliability of the OPCRIT system. *Archives of general psychiatry* 1991; **48**(8)**:** 764-770.

3. Berendsen S, Kapitein P, Schirmbeck F, van Tricht MJ, McGuire P, Morgan C *et al.* Pre-training inter-rater reliability of clinical instruments in an international psychosis research project. *Schizophrenia research* 2021; **230:** 104-107.

4. Gayer-Anderson C, Jongsma HE, Di Forti M, Quattrone D, Velthorst E, De Haan L *et al.* The European network of national schizophrenia networks studying gene–environment interactions (EU-GEI): Incidence and first-episode case–control programme. *Social psychiatry and psychiatric epidemiology* 2020; **55:** 645-657.

5. Gayer-Anderson C, Jongsma HE, Di Forti M, Quattrone D, Velthorst E, de Haan L *et al.* The EUropean Network of National Schizophrenia Networks Studying Gene-Environment Interactions (EU-GEI): Incidence and First-Episode Case-Control Programme. *Social Psychiatry & Psychiatric Epidemiology* 2020; **55**(5)**:** 645-657.

6. Brittain PJ, Lobo SEM, Rucker J, Amarasinghe M, Anilkumar APP, Baggaley M *et al.* Harnessing clinical psychiatric data with an electronic assessment tool (OPCRIT+): the utility of symptom dimensions. *Plos one* 2013; **8**(3)**:** e58790.

7. Brittain PJ, Stahl D, Rucker J, Kawadler J, Schumann G. A review of the reliability and validity of OPCRIT in relation to its use for the routine clinical assessment of mental health patients. *International journal of methods in psychiatric research* 2013; **22**(2)**:** 110-137.

8. Di Forti M, Quattrone D, Freeman TP, Tripoli G, Gayer-Anderson C, Quigley H *et al.* The contribution of cannabis use to variation in the incidence of psychotic disorder across Europe (EU-GEI): a multicentre case-control study. *The Lancet Psychiatry* 2019; **6**(5)**:** 427-436.

9. Fearon P, Kirkbride JB, Morgan C, Dazzan P, Morgan K, Lloyd T *et al.* Incidence of schizophrenia and other psychoses in ethnic minority groups: results from the MRC AESOP Study. *Psychol Med* 2006; **36**(11)**:** 1541-1550.

10. Jongsma HE, Gayer-Anderson C, Lasalvia A, Quattrone D, Mulè A, Szöke A *et al.* Treated Incidence of Psychotic Disorders in the Multinational EU-GEI Study. *JAMA psychiatry* 2018; **75**(1)**:** 36-46.

11. Rodriguez V, Alameda L, Quattrone D, Tripoli G, Gayer-Anderson C, Spinazzola E *et al.* Use of multiple polygenic risk scores for distinguishing schizophrenia-spectrum disorder and affective psychosis categories in a first-episode sample; the EU-GEI study. *Psychol Med* 2022**:** 1-10.
